# Supplementary material for: The causal relationship between the human gut microbiota and pyogenic arthritis: a Mendelian randomization study
Source: Front Cell Infect Microbiol. 2024 Nov 26;14:1452480. doi: 10.3389/fcimb.2024.1452480 (PMC11629706; doi:10.3389/fcimb.2024.1452480)
Supplement: Supplementary file 1 [file DataSheet1.zip › mendelian test/ebi-a-GCST90027816.csv_leave_one_out.pdf]

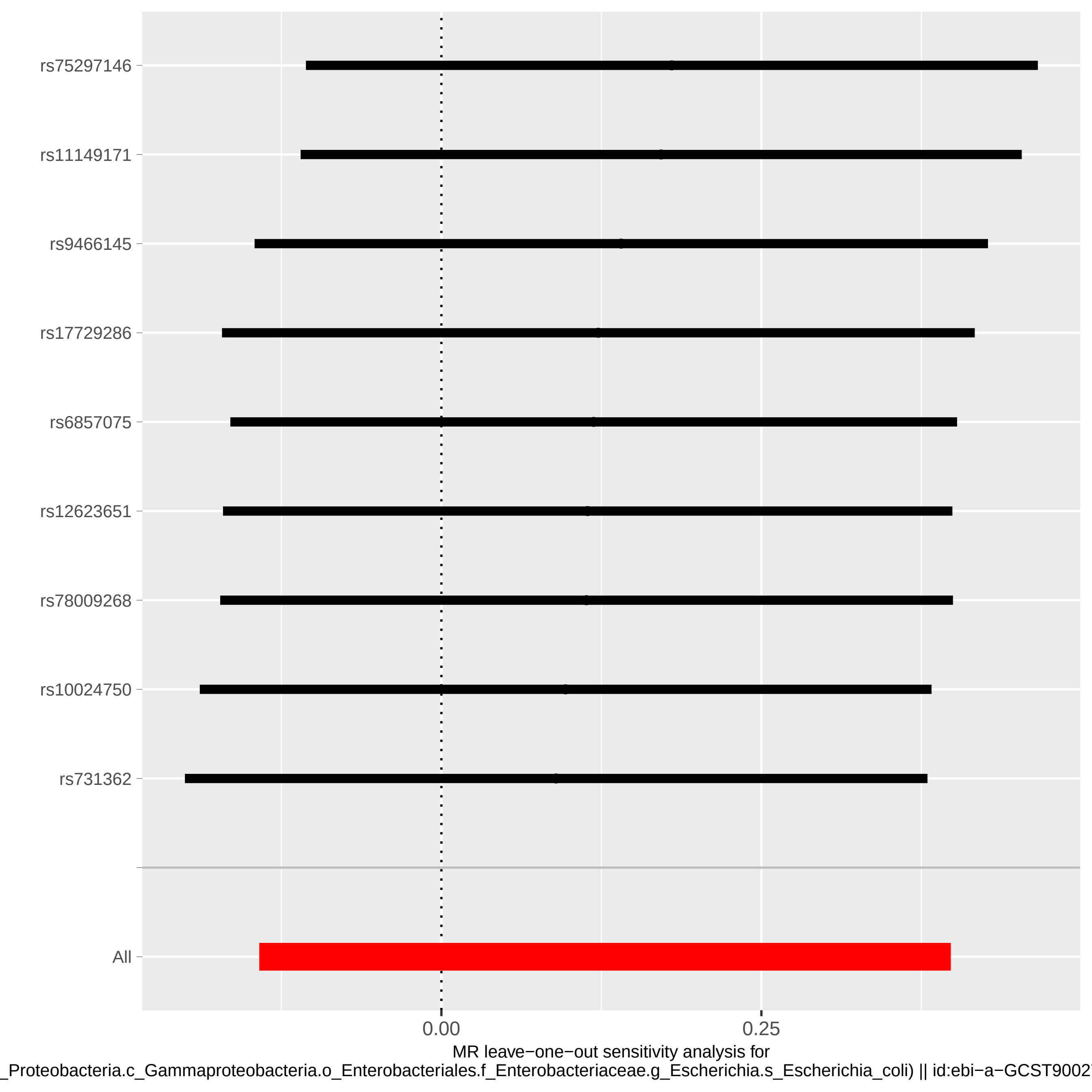
rs75297146
rs11149171
rs9466145
rs17729286
rs6857075
rs12623651
rs78009268
rs10024750
rs731362
All
0.00
0.25
MR leave−one−out sensitivity analysis for
_Proteobacteria.c_Gammaproteobacteria.o_Enterobacteriales.f_Enterobacteriaceae.g_Escherichia.s_Escherichia_coli) || id:ebi−a−GCST9002
